# Effectiveness of the Chest Pain Choice decision aid in emergency department patients with low-risk chest pain: study protocol for a multicenter randomized trial

**DOI:** 10.1186/1745-6215-15-166

**Published:** 2014-05-10

**Authors:** Ryan T Anderson, Victor M Montori, Nilay D Shah, Henry H Ting, Laurie J Pencille, Michel Demers, Jeffrey A Kline, Deborah B Diercks, Judd E Hollander, Carlos A Torres, Jason T Schaffer, Jeph Herrin, Megan Branda, Annie Leblanc, Erik P Hess

**Affiliations:** 1 Mayo Medical School, Mayo Clinic College of Medicine, 200 First Street SW, Rochester, MN 55905, USA; 2 Knowledge and Evaluation Research Unit, Mayo Clinic, 200 First Street SW, Rochester, MN 55905, USA; 3 Department of Internal Medicine, Division of Endocrinology, Mayo Clinic, 200 First Street SW, Rochester, MN 55905, USA; 4 Department of Health Sciences, Division of Healthcare Policy and Research, Mayo Clinic, 200 First Street SW, Rochester, MN 55905, USA; 5 Center for the Science of Healthcare Delivery, Mayo Clinic, 200 First Street SW, Rochester, MN 55905, USA; 6 Department of Internal Medicine, Division of Cardiology, Mayo Clinic, 200 First Street SW, Rochester, MN 55905, USA; 7 Patient representative, Rochester, MN, USA; 8 Department of Emergency Medicine, Indiana University School of Medicine, 720 Eskanazi Ave IN, Indianapolis, IN 46202, USA; 9 Department of Cellular and Integrative Physiology, Indiana University School of Medicine, 720 Eskanazi Ave IN, Indianapolis, IN 46202, USA; 10 Department of Emergency Medicine, University of California Davis Medical Center, 2315 Stockton Boulevard, Sacramento, CA 95817, USA; 11 Department of Emergency Medicine, University of Pennsylvania, 3400 Spruce Street, Philadelphia, PA 19104, USA; 12 Department of Emergency Medicine, Mayo Clinic, 4500 San Pablo Road South, Jacksonville, FL 32224, USA; 13 Department of Internal Medicine, Division of Cardiology, Yale University School of Medicine, 333 Cedar Street, New Haven, CT 06510, USA; 14 Health Research and Educational Trust, 1 North Franklin Street, Chicago, IL 60606, USA; 15 Caregiver Representative, Mayo Clinic, 200 First Street SW, Rochester, MN 55905, USA; 16 Department of Emergency Medicine, Division of Emergency Medicine Research, Mayo Clinic, 200 First Street Southwest, Rochester, MN 55905, USA

**Keywords:** Chest pain, Acute coronary syndrome, Acute myocardial infarction, Unstable angina, Shared decision-making, Emergency department, Healthcare utilization

## Abstract

**Background:**

Chest pain is the second most common reason patients visit emergency departments (EDs) and often results in very low-risk patients being admitted for prolonged observation and advanced cardiac testing. Shared decision-making, including educating patients regarding their 45-day risk for acute coronary syndrome (ACS) and management options, might safely decrease healthcare utilization.

**Methods/Design:**

This is a protocol for a multicenter practical patient-level randomized trial to compare an intervention group receiving a decision aid, Chest Pain Choice (CPC), to a control group receiving usual care. Adults presenting to five geographically and ethnically diverse EDs who are being considered for admission for observation and advanced cardiac testing will be eligible for enrollment. We will measure the effect of CPC on (1) patient knowledge regarding their 45-day risk for ACS and the available management options (primary outcome); (2) patient engagement in the decision-making process; (3) the degree of conflict patients experience related to feeling uninformed (decisional conflict); (4) patient and clinician satisfaction with the decision made; (5) the rate of major adverse cardiac events at 30 days; (6) the proportion of patients admitted for advanced cardiac testing; and (7) healthcare utilization. To assess these outcomes, we will administer patient and clinician surveys immediately after each clinical encounter, obtain video recordings of the patient-clinician discussion, administer a patient healthcare utilization diary, analyze hospital billing records, review the electronic medical record, and conduct telephone follow-up.

**Discussion:**

This multicenter trial will robustly assess the effectiveness of a decision aid on patient-centered outcomes, safety, and healthcare utilization in low-risk chest pain patients from a variety of geographically and ethnically diverse EDs.

**Trial registration:**

NCT01969240.

## Background

Chest pain is the second most common reason patients visit emergency departments (EDs) across the United States, accounting for over 8 million visits and 25% of hospital admissions annually [1,2]. Information from the history and physical examination, electrocardiogram, and initial cardiac troponin alone lack sufficient sensitivity to reliably eliminate patients without acute coronary syndrome (ACS) who are safe for ED discharge. As missed ACS may lead to an acute myocardial infarction (AMI) and/or potentially preventable death or disability, clinicians have a low threshold to hospitalize patients with chest pain. This results in increased psychological morbidity for patients - including anxiety and depression - and hospital overcrowding, increased false positive test results, unnecessary downstream procedures, and between 3 and 10 billion dollars spent annually for patients found not to have cardiac disease [3,4].

Several contemporary prediction rules for ED patients with possible ACS have been developed and validated [5-9]. To date, however, none have been widely adopted by clinicians and consistently translated into practice. A recent trial suggests that it may be feasible to discharge low-risk patients with possible cardiac chest pain from the ED without cardiac stress testing or a computed tomography (CT) coronary angiography [10]. However, ED patients being evaluated for possible ACS are rarely aware of their risk for an adverse cardiac event, [11] and in one recent trial lower patient satisfaction was observed in the intervention arm in which clinicians used evidence-based decision support without explicit risk communication and patient engagement [12].

In the context of emergency medicine, clinicians frequently practice under conditions of high patient volume and acuity and are often required to make decisions expeditiously to ensure the safety of patients waiting to be seen. The approach clinicians take to medical decision-making may vary based on any number of factors, such as the degree of patient acuity, the cognitive workload of the clinician at a particular moment in time, and their perception of a patient’s desire to engage in decisions regarding their care, among others. Moreover, the approach adopted at the onset of a medical encounter may change as the interaction evolves [13]. One way to think of this is as a fluid process, in which an experienced clinician seamlessly transitions from one decision-making model to the next, depending on the approach that seems most appropriate given the clinical context and the individual patient.

In prior work we developed a decision aid, Chest Pain Choice (CPC), [14] and tested the hypothesis that use of the decision aid during a clinical encounter would facilitate a shared approach to medical decision-making, improve patient-centered decision-based outcomes (patient knowledge, engagement in the decision-making process and satisfaction), and safely decrease resource utilization. The decision aid described to patients the rationale for their chest pain evaluation and the potential utility of urgent cardiac stress testing. It also included a precise estimate of the 45-day risk for ACS derived from a robustly validated prediction model [5,15,16] and communicated that risk using prose phrases, numbers, and a pictograph to account for the patient’s preferred mode of understanding numerical information [14]. In a single-center randomized pilot trial (n = 204) we observed greater knowledge regarding management options and the short-term risk for ACS, greater engagement in the decision-making process, and a 19% lower rate of observation unit admission for cardiac stress testing in patients randomized to this shared decision-making intervention compared to usual care, and there were no major adverse cardiac events within 30 days in either arm [11].

In this paper we describe the rationale and methods employed to test the effectiveness of the CPC decision aid in a multicenter trial in five geographically and ethnically diverse hospital EDs in the United States. We hypothesize that use of the decision aid will significantly increase patient knowledge, engagement and satisfaction, and decrease the rate of testing that may have marginal benefit in the low-risk population with no increase in adverse events.

## Methods/Design

This practical, multicenter randomized trial compares an intervention group receiving a structured risk assessment and corresponding decision aid (CPC), to a control group receiving usual care [17]. Institutional Review Board approval has been obtained at each participating institution. The trial is registered at clinicaltrials.gov (NCT01969240).

### Setting

Patients and physicians will be recruited from five EDs: the University of Pennsylvania in Pennsylvania, United States (east coast with an urban patient population); the Mayo Clinic in Rochester, Minnesota, United States (midwest with a rural patient population); the Mayo Clinic in Jacksonville, Florida, United States (southeast with an urban patient population); the University of California, Davis in California, United States (west coast with an urban patient population); and Indiana University Health Methodist Hospital in Indianapolis, Indiana, United States (midwest with an urban patient population). We selected these five settings to assess the effectiveness of the decision aid in geographically diverse clinical practice contexts and ethnically diverse patient populations. The trial will be conducted in the flow of routine patient care.

### Participants

#### Participant recruitment

**Figure 1:**
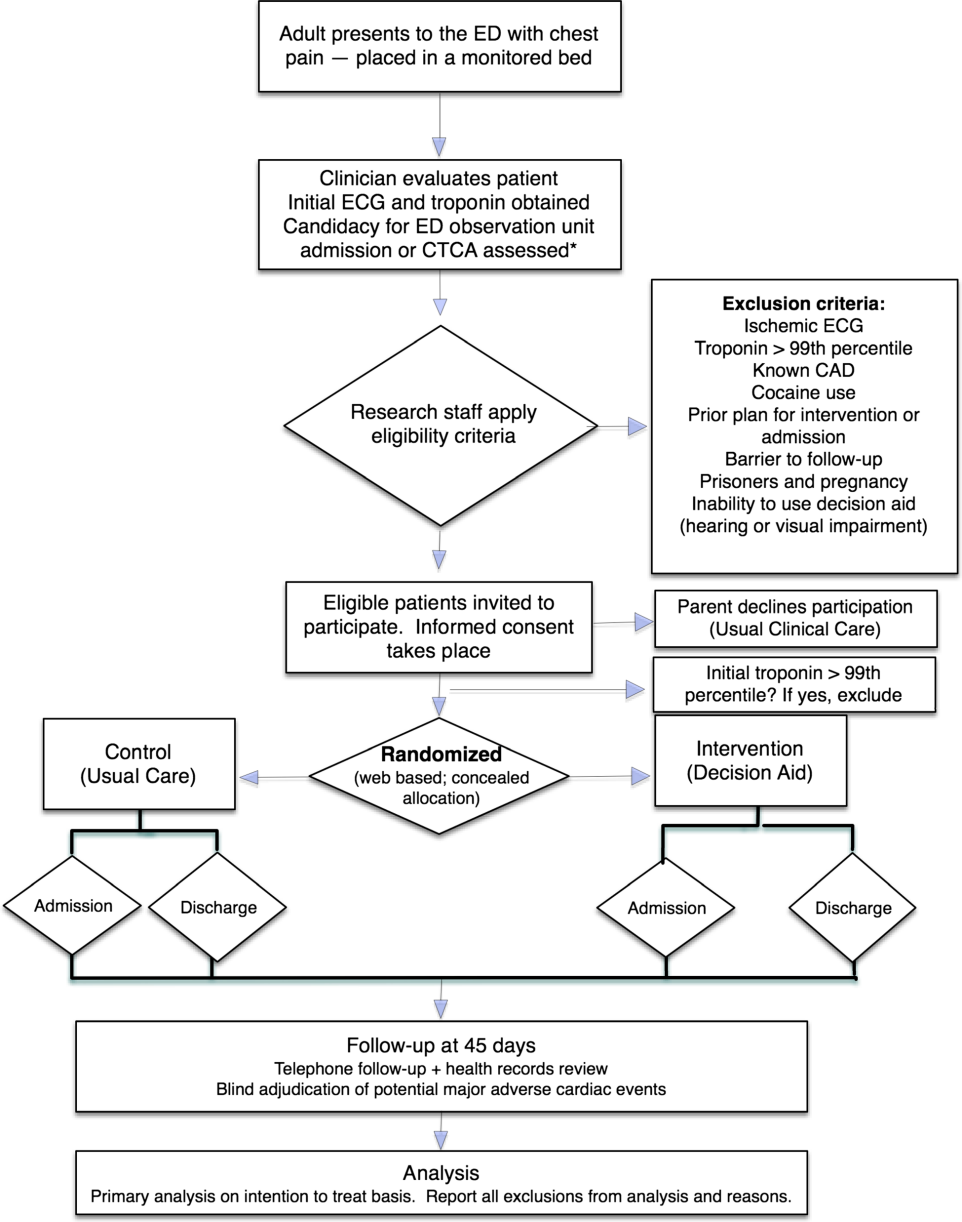
**Flow diagram showing the integration of study procedures in the flow of patient care.** *Candidacy for ED observation unit admission will be assessed after the results of the initial troponin are available in some cases. CTCA, computed tomographic coronary angiography; ED, emergency department; CAD, coronary artery disease; ECG, electrocardiogram.

A study coordinator will identify potentially eligible patients based on the chief complaint and, in collaboration with the treating clinician, confirm patient eligibility for the trial (Figure 1). The study coordinator will then obtain written informed consent from the patient. Consent will be obtained from participating clinicians before or at the time of patient consent. Patients will be consecutively enrolled at each of the participating centers six to seven days per week whenever a study coordinator is available for enrollment.

#### Eligibility criteria

Eligible clinicians will include ED physicians and mid-level providers caring for patients with chest pain. Eligible patients will include adults (>17 years of age) presenting to the ED with a chief complaint of chest pain who are being considered by the treating clinician for admission for additional observation and cardiac stress testing or for further cardiac testing with coronary CT angiography. Exclusion criteria are listed in Figure 1. Data will be collected on all patients assessed for eligibility, including the number of excluded patients, the rationale for each exclusion, and the number of patients who decline consent to participate in the study in accordance with the CONSORT guidelines for reporting randomized trials [18].

#### Integrating patient recruitment, consent, and delivery of the intervention in the flow of patient care

Figure 1 shows how the process of patient recruitment, consent, randomization, and delivery of the intervention will integrate with the flow of patient care. We will attempt to assess patient eligibility and obtain consent shortly after the clinician has evaluated the patient and interpreted the initial ECG, but before the result of the first cardiac troponin is available. Patients who are otherwise eligible for the study but in whom the first cardiac troponin result is greater than the 99th percentile reference limit will be excluded prior to randomization. Once the initial cardiac troponin result is determined to be negative, the patient will subsequently be randomized using a centralized web-based mechanism to either the decision aid or usual care arm. Patients who are randomized to the decision aid arm will then discuss with their clinician their 45-day risk for an adverse cardiac event and engage in shared decision-making. Patients randomized to usual care will receive no intervention. The number and timing of subsequent cardiac troponin measurements will not be prescribed by the study protocol. Instead patients will subsequently undergo serial cardiac troponin measurements to rule out AMI according to the institutional protocol at each participating site.

#### Patient and stakeholder engagement in the trial

Patients, their caregivers, and other key stakeholders were and will be engaged throughout the entire research process and will comprise the patient and stakeholder advisory group.

The emergency department Patient Advisory Council, which consists of five individuals who have contributed the patient’s perspective to several practice and quality improvement initiatives at Mayo Clinic Rochester - an ED quality specialist, three patients, and a nurse representative - provided feedback on the grant proposal and will continue to inform on the conduct of the trial and the interpretation of the results when the trial is complete. A patient and caregiver representative were engaged in the preparatory phases of research and assisted in the development and iterative refinement of the CPC decision aid, provided feedback on the grant proposal submitted for funding, selected the primary outcome for study, are active members on the investigative steering committee, and participate in the study as co-investigators. We will also engage the patient and caregiver representatives in the interpretation and dissemination of the study findings once the trial is complete.

#### Randomization

Patients will be randomized after informed consent is obtained and it is confirmed that all inclusion and no exclusion criteria are met, prior to the patient-clinician discussion of treatment options and patient risk. Allocation will be concealed by means of an online password-protected randomization algorithm designed using Medidata Balance™ Medidata Solutions, New York City, USA. Patients will be dynamically stratified [19] by enrolling by site, age and sex because of the known association of age and sex with cardiovascular risk and the availability of these data at the time of enrollment. Allocation will be based on a 1:1 ratio between the intervention and usual care arms. Though we will not be able to blind patients and clinicians to use of the decision aid, we will blind the investigators and individuals not involved in providing care to the patient allocation.

### Intervention arm

#### Refinement of the decision aid

**Figure 2:**
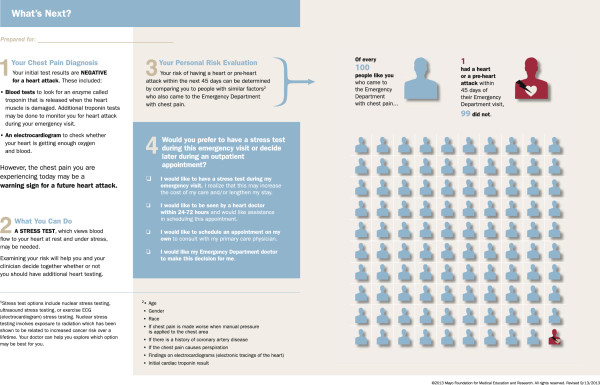
**The Decision Aid.** The decision aid describes for patients the rationale for, and results of, the initial emergency department evaluation (electrocardiogram, initial cardiac troponin level) and the potential utility of additional cardiac testing. A reliable estimate of the risk of an acute coronary syndrome within 45 days, obtained from a quantitative pretest probability (QPTP) web-tool developed and tested by Kline *et al*. [5,15,16], is included. The decision aid will be individualized to the patient based on the results of the QPTP risk calculator. The risk estimate is displayed using a state-of-the-art risk communication pictograph using an ordered icon array displaying natural frequencies, and a prose description of patient risk (for example, out of every 100 patients with factors like yours, 1 had a heart attack or pre-heart attack diagnosis within 45 days, 99 did not) to account for differences in numeracy preferences between patients. The decision aid also provides explicit management options (admission with urgent cardiac stress testing, follow-up with a cardiologist or the patient’s own primary care physician within 24 to 72 hours, or have the clinician make the decision on the patient’s behalf) for the clinician and patient to consider when reaching a shared decision.

The initial CPC decision aid was developed and tested in a single-center pilot randomized trial at the Mayo Clinic in Rochester, Minnesota, United States, as described previously [11]. The process of decision aid development was evidence-based, iterative, involved patients and key stakeholders, was based on participatory action research, used design approaches to ensure the final iteration met the needs of end-users, and resulted in a tool that satisfied International Patient Decision Aid Standards (IPDAS) [20]. After the pilot trial was completed and prior to beginning the multicenter trial, the decision aid underwent additional revisions based on input from the patient (MD) and caregiver (AL) representatives engaged in the trial and the investigative team in order to increase the clarity of the decision aid and improve patient comprehension. The refined CPC one-page decision aid is included in Figure 2.

**Figure 3:**
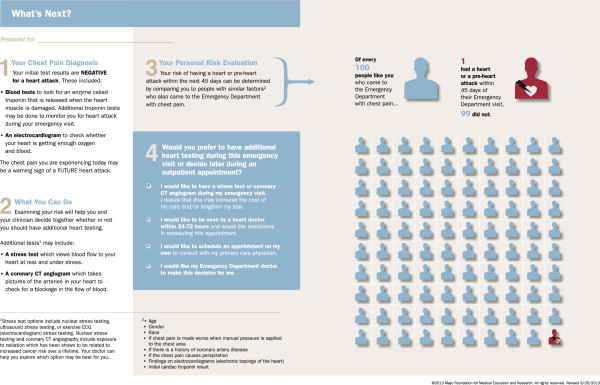
**Decision aid that includes the option of coronary CT angiography.** This version of the decision aid was developed for use at the University of Pennsylvania emergency department (Pennsylvania, United States) in which coronary CT angiography is frequently used in lieu of cardiac stress testing.

In the process of preparing for the trial it became clear that one of the participating sites (University of Pennsylvania) frequently used coronary CT angiography in chest pain patients at low-risk for ACS in lieu of cardiac stress testing. To ensure the decision aid facilitated a conversation that included options relevant to the setting in which patients are frequently considered for coronary CT angiography, a second version of the decision aid was developed for use at this site (Figure 3).

Participating clinicians were trained by means of a one-hour grand rounds presentation given by the lead investigator at each participating site, as well as a training video that demonstrated use of the decision aid in a simulated patient encounter. The lead study coordinator at the central coordinating site trained each of the study coordinators at the participating sites to ensure comprehension of the study protocol and the standardized execution of study procedures. As was done in our pilot trial, study coordinators will offer to provide the treating clinician a concise refresher of the content included in the decision aid at the time of patient enrollment. Videos of the patient-clinician disposition discussion will be obtained to assess the fidelity of the use of the decision aid in the intervention group and to monitor for contamination in the control group. Video and audio recordings will be consented to separately and are not required for participation in the trial.

#### Delivery of the intervention

For patients randomized to the decision aid arm, the study coordinator will collect each of the variables entered into the quantitative pretest probability (QPTP) PREtest consult Inc. Charlotte, USA calculator, ask the treating clinician to verify and sign off on their accuracy, generate the 45-day estimate of ACS risk, and select the decision aid corresponding to the appropriate level of risk. The study coordinator will then provide the clinician with a color copy of the decision aid prior to the disposition discussion with the patient and offer to provide a concise refresher of the content included in the decision aid. The treating clinician will then, using the decision aid as a tool to facilitate discussion, educate the patient regarding the rationale for their evaluation up to that point in the ED visit, communicate the 45-day risk for a heart attack or pre-heart attack, and engage the patient in a shared decision regarding whether to obtain further cardiac testing after AMI has been ruled out by cardiac troponin testing, to follow up as an outpatient within 72 hours of the ED visit with a cardiologist or their primary care physician, or to have the emergency clinician decide on the patient’s behalf.

#### Usual care

For patients randomized to the usual care arm the clinician will discuss the results of diagnostic investigations and management options with the patient in that clinician’s usual fashion. The patient’s risk will be calculated using the QPTP calculator for study purposes, however, the patient and clinician will be blinded to the QPTP-structured risk assessment and the corresponding decision aid in this arm. As in the intervention arm, the clinician-patient disposition discussion will be video and audio recorded to assess the degree to which the clinician engages the patient in the decision-making process and to monitor for contamination in the control arm.

#### Outcome measures

When selecting outcome measures we obtained input from a patient and caregiver representative, clinicians (emergency medicine clinicians and cardiologists), researchers (health services researchers and shared decision-making experts), and a payer representative (Chief Medical Officer of Mayo Clinic Health Solutions). In face-to-face meetings with the patient and caregiver representative it became clear that knowledge was the outcome that was of greatest interest to the patient, so patient knowledge was selected as the primary outcome. Thus, as we did in our pilot trial, we will assess patient’s knowledge regarding their short-term risk for ACS, the potential implications of cardiac testing, the available management options, and the potential risk of radiation exposure through a post-visit survey administered immediately after the clinical encounter [11].

We will also assess a number of secondary outcomes, including patient engagement in the decision-making process, as measured by the OPTION (observing patient involvement) scale [21]; the degree of conflict patients experience related to feeling uninformed, as measured by the Decisional Conflict Scale (DCS) [22]; trust in the physician measured via the Trust in Physician Scale (TPS) [23]; patient satisfaction with the decision made, via a Likert scale on the post-visit survey; and safety as determined by the prevalence of Major Adverse Cardiac Events (MACE) within 30 days of the ED visit.

Consistent with recommendations in a recently published consensus document on ACS research in emergency departments [24], we will define MACE as AMI [25], death due to a cardiac or unknown cause, emergency revascularization, ventricular arrhythmia, or cardiogenic shock. We will exclude MACE that occurs during the index admission to the ED but rather focus on events occurring after discharge, in the outpatient setting, which could have potentially been avoided or diagnosed in the ED or hospital. This approach avoids having to adjudicate whether MACE occurring in the ED or hospital were ‘missed’ based on a post-hoc review of potentially incomplete medical records. We will collect data on all MACE that occur up to 45 days from the index ED visit as this is the follow-up interval assessed by the QPTP instrument [5,15,16]. However, we will compare rates only for events that occur prior to or at 30 days in order to comply with standardized reporting guidelines for risk stratification of ED patients with possible ACS and to facilitate comparison with other ACS risk stratification studies in the extant literature [26]. A subsequent analysis for all 45 days is planned, with these data to be included as part of an Additional file.

To account for differences in troponin assays between institutions, and for the potential of changing from one assay to another in a given institution during the trial, we will track the specific cardiac troponin assay used at each institution along with that assay’s characteristics (lower limit of detection, 99th percentile reference limit, and 10% coefficient of variation), the absolute value of each troponin measurement, the upper limit of normal used at that institution, and classify whether that value is above the upper limit of normal in each enrolled patient (Additional file 1). Three investigators blinded to the study arm will adjudicate all potential MACE cases and discordances will be noted and resolved by consensus.

One of the goals of this study is to measure the effect of CPC on healthcare utilization. Healthcare utilization will be assessed by measuring the proportion of patients admitted for cardiac testing, the most immediate clinical decision and the greatest driver of utilization, and the patients’ healthcare utilization for the subsequent 30 days after the ED encounter. This will include measures such as hospitalization, re-hospitalization, primary, specialty, and ED visits, and diagnostic and laboratory testing.

#### Data collection

Data documenting the process of screening for potentially eligible participants, application of the eligibility criteria (including the reason for each exclusion), the variables entered into the pretest probability calculator, the arm to which each patient is randomized, and other information best collected in real time will be recorded by study coordinators using a standardized case report form (Additional file 2). Patients’ preferences for understanding numerical information and their preferred approach to participation in medical decision-making will be assessed by a survey administered prior to the clinical encounter (Additional file 3).

Immediate post-visit surveys will collect data on patient knowledge, decisional conflict, patients’ trust in their physician, and patient satisfaction with the decision made (Additional file 4). The degree to which clinicians engage patients in the decision-making process will be assessed by having two trained team members independently review videos of the clinician-patient disposition discussion and apply the OPTION scale [21].

The patient will be contacted 45 days after enrollment via telephone (primary method of contact) for assessment of safety. If the primary method of contact is not successful the study coordinator will subsequently contact the patient utilizing a secondary method of contact specified by the patient at the time of consent (such as email, a secondary phone number, or mail) for a follow-up assessment. Safety will be assessed using a standardized phone follow-up script to determine whether a MACE occurred and, if so, the date of the event (Additional file 5). Events occurring within 45 days will be recorded to ensure consistency with the duration of follow-up used in the development of the QPTP instrument [15] and MACE within 30 days will be reported in accordance with standardized reporting guidelines for risk stratification studies of ED patients with potential ACS [26].

Utilization data will be collected from the electronic medical record at each site, patient self-report, and UB-92 and UB-04 summary hospital billing statements. Whether the patient was admitted to the observation unit or hospital and/or underwent advanced cardiac testing such as cardiac stress testing, coronary CT angiography, or percutaneous coronary angiography, will be assessed by review of the electronic medical record at each institution. Patients will be given a healthcare utilization diary at the time of ED discharge (Additional file 6) and educated regarding its use to facilitate the standardized collection of healthcare utilization data not included in hospital billing statements. Any entries in the healthcare diary, including the date and location of service, will be ascertained at the time of 45-day follow-up. Healthcare utilization within 30 days of the ED visit will be reported for study purposes.

Clinicians will be surveyed immediately after each encounter to determine their perception of the degree of patient involvement in the decision, the helpfulness of the information provided to the patient, and other characteristics of the decision-making process (Additional file 7).

Study data will be collected and managed using REDCap (Research Electronic Data Capture) tools hosted at the Mayo Clinic [27]. REDCap is a secure, web-based application designed to support data capture for research studies, providing: 1) an intuitive interface for validated data entry; 2) audit trails for tracking data manipulation and export procedures; and 3) automated export procedures for seamless data downloads to common statistical packages.

An independent data and safety monitoring board (DSMB) will monitor safety, scientific, and ethical aspects of the study. The DSMB met prior to beginning enrollment and will meet every six months throughout the duration of the study. Though every effort will be made to minimize post-randomization exclusions, patients will be excluded after randomization if they are found to meet exclusion criteria that were not recognized at the time of enrollment or they choose to withdraw [28]. Any potential MACE following discharge from the ED or hospital will be reported to the principal investigator and the DSMB. The authority to stop the study will be retained by the DSMB and must be based on consensus.

#### Statistical considerations

In order for the study findings to maximally impact practice and policy, they will need to be adequately powered to detect differences in outcomes of interest to each of the stakeholders (patients, clinicians, shared decision-making research experts, and healthcare payers). We will have sufficient funding to enroll 930 patients, accounting for a loss to follow-up rate as high as 5% (there was 2% lost to follow-up in our pilot trial) [11], we anticipate having complete data on 884 patients. Table 1 shows the anticipated power we will have to detect differences in each of the patient and stakeholder-important outcomes on completion of the trial.

**Table 1:** Anticipated power to detect patient and stakeholder-important outcomes on completion of the trial

**Outcome (n = 884)**	Usual Care*	Decision Aid*	**Difference**	**Power**
**Patient knowledge**	44% (23.3)	60% (20.9)	16% (9.5%, 22.5%)	99%
**Patient engagement in the decision-making process (n = 221)**	7.0 (5.5)	26.3 (8.2)	19.3 (18.4, 20.2)	99%
**Decisional conflict†**	35.9 (18.9)	22.3 (21.1)	13.6 (11.0, 16.2)	99%
**Trust in the physician**	79.3 (19.9)	83.4 (19.8)	4.1 (1.5, 6.7)	86%
**Patient satisfaction with the decision made (% agree or strongly agree they are satisfied)**	69.7 (25.6)	80 (25.6)	10.3 (4.6%, 16.0%)	99%
**Safety (major adverse cardiovascular events)‡**	0%	0%	0% (−−)	78%
**Proportion of patients admitted for cardiac testing**	77%	67%	10% (4.1, 15.9)	90%
**Healthcare utilization**	8.3 (0.8)	7.0 (0.7)	1.3 (1.2, 1.4)	99%

*Estimates were determined from our completed pilot randomized trial [11].

†Lower decisional conflict scores indicate less conflict experienced by patients related to feeling uninformed.

‡Non-inferiority, one-sided test with alpha = 0.05 with a maximum difference of 5%.

We will use standard techniques appropriate for patient-randomized trials, with each outcome compared between study arms using t-tests for continuous outcomes and chi-square tests for dichotomous outcomes. Rather than assume the intervention effect is independent of the study site where the patient is seen or of the clinician who sees them, we will assess intra-site and intra-clinician correlations. We will then stratify all tests on the study site unless the intra-clinician correlation is non-trivial, in which case we will stratify on a clinician basis. We will calculate differences and their confidence intervals using standard techniques for continuous and dichotomous data. If there are differences in baseline characteristics between the two study groups, these will be accounted for using regression models that will include an indicator for study arm. Finally, we will analyze clinician surveys using regression models with robust standard errors to account for the repeated measures on clinicians.

Descriptive analyses will be performed to describe any potential heterogeneity of treatment effect (HTE) and facilitate synthesis of subgroup results in future meta-analyses. We will conduct descriptive HTE analyses by age, gender, race/ethnicity, quantitative pre-test probability of ACS, level of education, OPTION scores, and study site. The outcomes assessed with HTE analyses will be the same as those assessed in the trial (such as knowledge, engagement, satisfaction, and healthcare utilization). We will also conduct interaction testing to determine the interaction between the decision aid and each pre-specified characteristic.

Resource use will be compared both descriptively and using multivariable models. Descriptive comparison for 30-day utilization will utilize the Wilcoxon rank-sum test to account for the skewness of the outcome measures. The multivariable models for resource utilization will be estimated using the two-part or one-part negative binomial or log gamma regression models. A two-part model will be utilized for outcomes where more than 10% of the subjects have a zero outcome measure. The covariates in the multivariable models will include factors that may not be accounted for in the randomization such as comorbidities and severity of illness. Comorbidities will be measured using the Elixhauser method, which is a commonly used measure for the acute setting [29]. We will also analyze the results using a subset of patients who primarily receive their entire healthcare at the participating centers. We expect that this analysis will provide a sensitivity analysis of the overall trial results based on self-report.

## Discussion

We have described the rationale and methodology for a multicenter randomized clinical trial to measure the effectiveness of a decision aid on validated patient-centered outcome measures, safety, and healthcare utilization in ED chest pain patients at low-risk for ACS. The trial represents a dynamic and ongoing collaboration between a patient and caregiver representative, the ED patient advisory council at Saint Mary’s Hospital in Rochester (Minnesota, United States), ED clinicians, shared decision-making experts, a cardiologist, and health services researchers and will be the first multicenter shared decision-making trial conducted in an ED setting. If findings from our single-centered randomized trial [11] are externally validated in a variety of hospital EDs, it will also serve as proof of concept that can subsequently be applied to other emergency conditions and lay the groundwork for future implementation studies to explore how to best incorporate shared decision-making as part of routine clinical care in the emergency setting.

An important reason to conduct feasibility trials of decision aids designed for use in the clinical setting is that the tools have potential to impact the nature and content of the patient-clinician discussion as well as the duration of the clinical evaluation. Although it was not a significant barrier to patient engagement in our pilot trial, patients who present to the ED with chest pain may be in a vulnerable emotional state such that they might prefer decisions be made on their behalf rather than actively participate in their medical care. The ED is also the safety net for patients of all levels of education and for those without medical insurance, and we anticipate that there may be knowledge transfer challenges unique to this practice setting. When clinicians with different practice patterns and levels of experience use the decision aid they may not find it appropriate to use for some patients, or may not use the decision aid appropriately. Therefore, video recording the patient-clinician encounter may provide insight into these potential challenges, the effectiveness of the decision aid, and the nature of the patient-clinician interaction in a busy ED environment.

Randomizing at the patient level increases the risk of contamination between the intervention and control arms. We have taken several steps to minimize the risk of contamination. First, the 45-day ACS risk calculator is password protected and clinicians will not be able to determine an individual patient’s risk of ACS using this instrument, or have access to the decision aid [16]. Furthermore, in our pilot trial, we observed that when clinicians did not have access to the decision aid and patients’ 45-day risk of ACS, they reverted back to their usual practice of rapid decision-making and limited discussion with patients and were reticent to engage patients in shared decision-making. Second, only the site coordinators will have access to copies of the decision aid, and clinicians will not be provided the decision aid in the usual care arm. Third, site coordinators have been carefully trained to confirm the accuracy of each of the variables needed to generate the 45-day ACS risk estimate by review and sign-off by the treating clinician, but are instructed to keep the clinician blind to a patient’s risk unless a patient is randomized to the decision aid. Fourth, every effort will be made to obtain video and audio data of the disposition discussion in both the intervention and control arms, and we will monitor for contamination by review of these data. Finally, if contamination does occur, we anticipate it will decrease the magnitude of differences observed in the intervention and control groups, biasing the results towards the null.

There are advantages and disadvantages to using two different decision aids in the context of this trial. Having a separate decision aid (Figure 2) for the site in which coronary CT angiography is routinely used in lieu of cardiac stress testing in low-risk chest pain patients introduces heterogeneity in the intervention. However, a key aspect of contemporary evidence-based medicine involves translating evidence into practice in a manner that is sensitive to local practice context [30] as well as patients’ values and preferences [31], and our decision to design two different decision aids reflects an underlying commitment to these values. In addition, a key goal of the decision aid is to generate a conversation about the evidence surrounding a management decision and, by necessity, the conversation must include the management options frequently used in that practice context to be relevant for participating clinicians. Finally, we plan to measure the effect of the intervention both overall and by site, which will provide insight into any differences in effectiveness between sites.

It is possible that not all of the clinicians at each site will be willing to participate in the trial. This has the potential to decrease the rate of recruitment. However, in our pilot trial over 90% of the clinicians at our site agreed to participate [11], and the majority of the sites participating in the trial have a track record of successfully recruiting ED chest pain patients for prospective research, therefore we anticipate a high degree of clinician support and participation in the study.

In conclusion, this trial will compare the effectiveness of a shared decision-making approach to usual care in patients with low-risk chest pain presenting to the ED for evaluation. Such an approach has the potential to safely tailor advanced cardiac testing to the degree of patient risk in a manner that educates and empowers patients to participate in the decision-making process, is consistent with patients’ values and preferences, and is supportive of clinicians’ knowledge and expertise.

## Trial status

The study began enrolling patients in October 2013.

## Abbreviations

ACS: Acute coronary syndrome; AMI: acute myocardial infarction; CAD: coronary artery disease; CPC: chest pain choice; CT: computed tomography; DSMB: data safety monitoring board; ECG: electrocardiography; ED: Emergency department; EM: Emergency medicine; THE: heterogeneity of treatment effect; MACE: Major adverse cardiac events; OPTION: observing patient involvement; QPTP: Quantitative pretest probability; REDCap: Research electronic data capture; UB: universal billing.

## Competing interests

Dr. Kline has stock ownership in CP Diagnostics LLC. None of the other authors have any competing interests to declare.

## Authors’ contributions

RA drafted and provided critical revisions to the manuscript and contributed to the conception and design of the study. VM provided critical revisions to the manuscript and contributed to the conception and design of the study by defining key study outcomes and providing insight into how to meaningfully engage the patient, caregiver, and ED patient advisory council in the study. NS contributed to the conception and design of the study by developing the healthcare utilization analysis, drafted sections of the manuscript, and provided critical revisions the manuscript. HT contributed to the conception and design of the study by defining key study outcomes and drafted and provided critical revisions to the manuscript. LP contributed to the conception and design of the study by developing the data collection instruments and provided critical revisions to the manuscript. MD contributed to the conception and design of the study by selecting the primary outcome, providing the patient perspective in refinement of the decision aid, and provided critical revisions to the manuscript. JK contributed to the conception and design of the study by development and refinement of the decision aid and provided critical revisions to the manuscript. DD contributed to the conception and design of the study and provided critical revisions to the manuscript. JEH contributed to the conception and design of the study by defining key study outcomes and provided critical revisions to the manuscript. CT contributed to the conception and design of the study and provided critical revisions to the manuscript. JS contributed to the conception and design of the study and provided critical revisions to the manuscript. JH contributed to the conception and design of the study by developing the statistical analysis plan, drafting portions of the manuscript, and provided critical revisions to the manuscript. MB contributed to the conception and design of the study by development of the web-based centralized randomization method, development of the database, and provided critical revisions to the manuscript. AL contributed to the conception and design of the study by selecting the primary outcome, provided the caregiver perspective in refining the decision aid, and provided critical revisions to the manuscript. EH: conceived and designed the study, obtained funding, drafted sections of the manuscript, and provided critical revisions to the manuscript. All authors read and approved the final manuscript.

## Supplementary Material

Additional file 1 Test Characteristics of the troponin assays currently used at 5 the hospital emergency departments participating in the Chest Pain Choice Trial. Click here for file

Additional file 2 Chest Pain Choice: case report form. Click here for file

Additional file 3 Making wiser choices about Chest Pain: pre encounter survey. Click here for file

Additional file 4 Making Wiser Choices about Chest Pain: post encounter survey. Click here for file

Additional file 5 Chest Pain Choice Trial: 45-day follow-up. Click here for file

Additional file 6 Chest Pain Study: use of healthcare services diary. Click here for file

Additional file 7 Making wiser choices about Chest Pain: clinician post encounter survey. Click here for file
