# Supplementary material for: Effectiveness of the Chest Pain Choice decision aid in emergency department patients with low-risk chest pain: study protocol for a multicenter randomized trial
Source: Trials. 2014 May 10;15:166. doi: 10.1186/1745-6215-15-166 (PMC4031497; doi:10.1186/1745-6215-15-166)
Supplement: Additional file 1 — Test Characteristics of the troponin assays currently used at 5 the hospital emergency departments participating in the Chest Pain Choice Trial. [file 1745-6215-15-166-S1.docx]

**Test Characteristics of the troponin assays currently used at 5 the hospital emergency departments participating in the Chest Pain Choice Trial**

**Hospital of the University of Pennsylvania**

Abbott i-Stat Troponin I

LLD: 0.02 ng/mL

99th percentile reference limit: 0.08 ng/mL

10% coefficient of variation: 0.10 ng/mL

level considered "elevated/abnormal": >0.08 ng/mL

**Indiana University**

Abbot i-stat Troponin I

LLD: 0.02 ng/mL

99^th^ percentile reference limit: 0.08 ng/mL

10% coefficient of variation: 0.10 ng/mL

level considered elevated/abnormal:>0.08 ng/mL

**Mayo Clinic Jacksonville**

Roche Elecsys troponin T

LLD: 0.01 ng/mL

99^th^ percentile of reference limit: 0.01 ng/mL

10% coefficient of variation: 0.03 ng/mL

level considered elevated/abnormal: ≥0.01ng/mL

**Mayo Clinic Rochester**

Roche Elecsys troponin T

LLD: 0.01 ng/mL

99^th^ percentile of reference limit: 0.01 ng/mL

10% coefficient of variation: 0.03 ng/mL

level considered elevated/abnormal: ≥0.01ng/mL

**University of California Davis**

Siemens Healthcare Diagnostics Advia Centaur XP Troponin I

LLD: 0.006 ng/mL

99^th^ percentile reference limit: 0.04ng/mL

10% coefficient of variation: 0.3ng/mL

level considered elevated/abnormal: ≥0.05 ng/mL
