# Supplementary material for: Effectiveness of the Chest Pain Choice decision aid in emergency department patients with low-risk chest pain: study protocol for a multicenter randomized trial
Source: Trials. 2014 May 10;15:166. doi: 10.1186/1745-6215-15-166 (PMC4031497; doi:10.1186/1745-6215-15-166)
Supplement: Additional file 2 — Chest Pain Choice: case report form. [file 1745-6215-15-166-S2.pdf]

Chest Pain Choice: Case Report Form

Initial Patient Screening

Date: ____________________

Study ID: ____________________ Staff Initials: ____________________

Inclusion Criteria:

	Yes	No
Adult (> 17 years of age) with a primary complaint of chest pain	☐	☐
Treating clinician's next consideration is hospital or observation unit admission for cardiac testing to evaluate possible ACS (or coronary CT angiography)	☐	☐

Exclusion Criteria:

	Yes	No
Ischemic changes on the ECG not known to be old, determined by the treating clinician *	☐	☐
Initial cardiac troponin T value > 99th percentile (≥ 0.01 ng/mL) **	☐	☐
History of coronary artery disease (for coronary revascularization procedure within previous 30 days)	☐	☐
Cocaine use within 72 hours by the clinician's initial history	☐	☐
Pregnancy	☐	☐
Referral to the emergency department by a physician for admission	☐	☐
Patient indicates that another hospital is his or her "hospital of choice" in the event of a return ED visit ***	☐	☐
Patient is undergoing medical clearance for a detox center or any involuntary court or magistrate order	☐	☐
Homelessness, out-of-town residence or other condition known to preclude follow-up	☐	☐
Patient is in police custody or currently incarcerated	☐	☐
Patient cannot read English or have, in their clinicians' judgment, major learning barriers such as visual or hearing impairment or dementia that would compromise their ability to give written informed consent (or use the decision aid) ****	☐	☐

** If there are ischemic changes on the ECG, check with the treating clinician for determination of whether the changes are old/new.*
*** If patient meets all other eligibility criteria but troponin results not yet received, patient consent may be pursued. Eligibility contingent upon troponin result.*
**** Does not need to be solicited.*
***** If English is not the patient's primary language, or patient has a visual or hearing impairment, or patient has dementia, obtain clinicians' judgment as to patient's capacity to participate, prior to pursuing consent.*

	Yes	No
Does patient meet initial eligibility criteria?	☐	☐
After patient meets initial eligibility screen, is the first troponin positive? If yes, record in REDCap as Screen Failure.	☐	☐

Quantitative Pretest Probability (QTPT) Variables

Age Group:
- ☐ < 35
- ☐ 35 – 38
- ☐ 39 – 50
- ☐ > 50

Race:
- ☐ African American
- ☐ Asian
- ☐ Hispanic
- ☐ White
- ☐ Other

Gender:
- ☐ Male
- ☐ Female

	Yes	No
Chest Pain Reproduced by Palpitation:	☐	☐
History of Coronary Artery Disease:	☐	☐
Diaphoresis:	☐	☐
EKG ST Depression > 0.5 mm:	☐	☐
T-Wave Inversion Deeper Than - 0.5 mm:	☐	☐

Clinician Sign-off

Printed Name of Clinician: ______________________________

Signature of Treating Clinician: ____________________________ Date: ______________

Level of Training:
- ☐ Resident
  - If resident, year of training (circle one): 1 2 3 4
- ☐ Fellow (completed EM residency)
- ☐ Advanced practice provider
- ☐ Attending EM physician

Patient Contact Information

Medical Record #: ______________________

First Name: ______________________

Middle Initial: ______________________

Last Name: ______________________

Date of Birth: ______________________

Ethnicity:
- ☐ Hispanic or Latino
- ☐ Not Hispanic or Latino

Race:
- ☐ American Indian/Alaska Native
- ☐ Asian
- ☐ Black or African American
- ☐ Native Hawaiian or other Pacific Islander
- ☐ White/Caucasian
- ☐ Other

Patient's Preferred Means of Contact:
- ☐ Phone
- ☐ Mail
- ☐ E-mail

Primary Phone Number (cell or other): ______________

Secondary Phone Number (cell or other): ______________

E-mail Address: ______________________________

Home Address: ____________________ City: ______________ State: ____ Zip: ______

Insurance (the patient's primary insurance payer for ED visit):
- ☐ Government (Medicare, Medicaid, Veteran's Health Administration, DOD)
- ☐ Commercial (all fee for the service carrier's and PPO's)
- ☐ HMO (coverage that provides healthcare services for members on a prepaid basis)
- ☐ None

Cardiac Risk Factors

	Yes	No	PR*	MR**
Hypertension?	☐	☐		
Hypertension source			☐	☐
Diabetes?	☐	☐		
Diabetes source			☐	☐
Family history of cardiac disease (direct blood male relative < 55, or female < 65 with ongoing, acute MI, sudden cardiac death, CABG, or PCI)?	☐	☐		
Family history of cardiac disease source			☐	☐
Dyslipidemia?	☐	☐		
Dyslipidemia source?			☐	☐
Renal insufficiency (creatinine > 2.5 mg/dL for men; > 2.0 mg/dL for women)?	☐	☐		
Renal insufficiency source			☐	☐
Smoker (current, recent cessation, former)?	☐	☐		

Smoking type:
- ☐ Current (smoking cigarettes within 1 month of ED visit)
- ☐ Recent cessation (stopped smoking cigarettes within 1 month and 1 year of ED visit)
- ☐ Former (stopped smoking cigarettes > 1 year before ED visit)

***PR:** Patient self-reported only*

****MR:** Medical record*

Cardiovascular History

	Yes	No	PR*	MR**
Angina?	☐	☐		
Angina source			☐	☐
Congestive heart failure?	☐	☐		
Congestive heart failure source			☐	☐
Cardiac arrest?	☐	☐		
Cardiac arrest source			☐	☐
Prior ventricular arrhythmia (Vtach or VF requiring cardioversion or anti arrythmics)?	☐	☐		
Prior ventricular arrhythmia (ventricular tachycardia) source			☐	☐
Prior atrial arrhythmia (atrial fibrillation/flutter or SVT)?	☐	☐		
Arial arrhythmia source			☐	☐
History of stroke or TIA (transient ischemic attack)?	☐	☐		
History of stroke or TIA source			☐	☐
Peripheral arterial disease (claudication, amputation for arterial vascular insufficiency, peripheral arterial bypass, aortic aneurysm, ankle brachial index > 0.8)?	☐	☐		
Peripheral arterial disease source			☐	☐
Rheumatoid arthritis?	☐	☐		
Rheumatoid arthritis source			☐	☐
Pulmonary embolism?	☐	☐		
Pulmonary embolism source			☐	☐

***PR:** Patient self-reported only*

****MR:** Medical record*

Disposition Encounter

What arm was the patient randomized to?

- ☐ Usual Care
- ☐ Decision Aid

Was a companion present?

- ☐ No
- ☐ Yes – Relationship unknown
- ☐ Yes – Family member
- ☐ Yes – Caregiver
- ☐ Yes – Friend

Was the encounter recorded?

- ☐ No
- ☐ Yes – Video
- ☐ Yes – Audio only

Additional Variables

Chest pain duration (of longest episode): ____________ minutes

Surveys

When was Pre Survey completed?

- ☐ Prior to discussion with treating clinician
- ☐ After discussion with treating clinician

Treating Clinician Responses:

What were the factors that influenced your decision to obtain further cardiac testing? ______________________

____

What was the main factor that influenced your decision today? ______________________

____

Patient in the Decision Aid Arm Responses:

What were the factors that influenced your decision to obtain further cardiac testing? ______________________

____

What was the main factor that influenced your decision today? ______________________

____

Values Provided by QPTP

45 Day ACS Pretest Probability	________	(provided by QPTP)
Number of ACS Outcomes?	________	
Number of Matched Patients?	________	
95% CI – Lower Bound	________	(enter the smaller number as a percent)
95% CI – Upper Bound	________	(enter the larger number as a percent)
Post Test Probability (after initial troponin)	________	

Lab Results

	Pos.	Neg.		
First troponin T	☐	☐	First troponin value:	____________
Second troponin T	☐	☐	Second troponin value:	____________
Third troponin T	☐	☐	Third troponin value:	____________

Systolic blood pressure, mmHg ____________ (first)

Diastolic blood pressure, mmHg ____________ (first)

Sa02% ____________
