# Supplementary material for: Effectiveness of the Chest Pain Choice decision aid in emergency department patients with low-risk chest pain: study protocol for a multicenter randomized trial
Source: Trials. 2014 May 10;15:166. doi: 10.1186/1745-6215-15-166 (PMC4031497; doi:10.1186/1745-6215-15-166)
Supplement: Additional file 3 — Making wiser choices about Chest Pain: pre encounter survey. [file 1745-6215-15-166-S3.docx]

Patient ID:

Making Wiser Choices about Chest Pain

Pre Encounter Survey


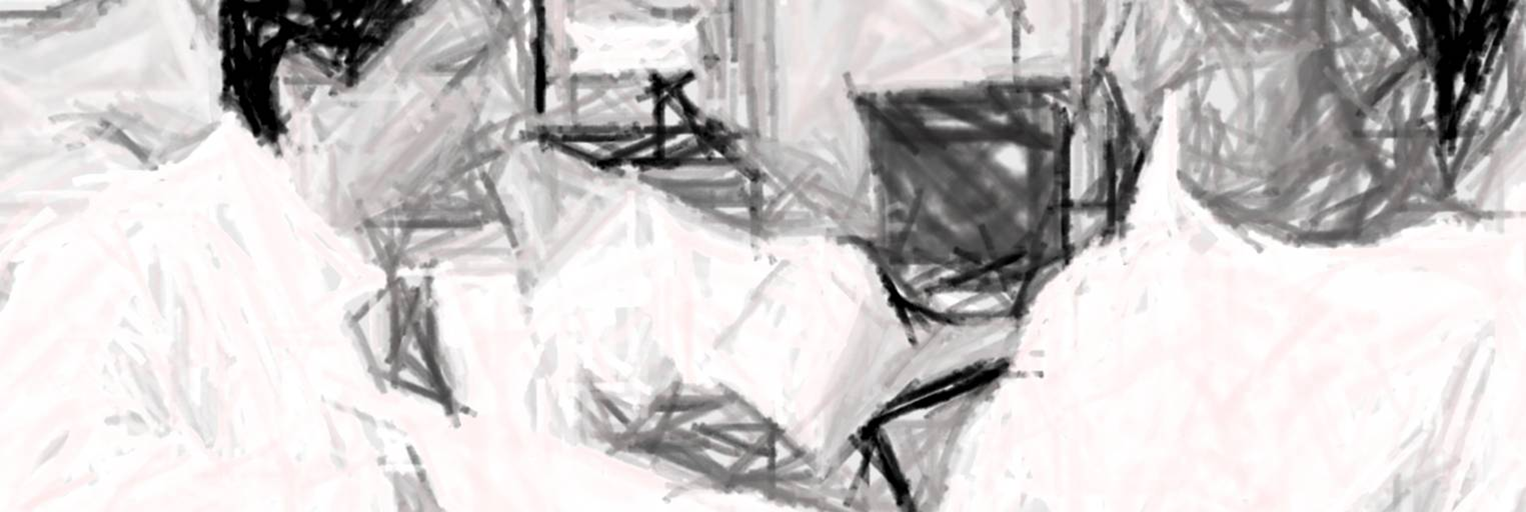


Patient Survey

Today’s Date: _ _/ _ _ / _ _ _ _

Month Day Year

Thank you for helping with this study. Your answers are very important to us. Please take the time to read and answer each question. Your responses are confidential and your clinician will not see your answers.

1. **For each of the following questions, please check the box that best reflects how good you are at doing the following things?**

| **How good are you at. . . . .** | **Not good**  **at all** | |  |  |  |  | **Extremely**  **good** | |
| --- | --- | --- | --- | --- | --- | --- | --- | --- |
| a. working with fractions? | |  |  |  |  |  | |  |
|  | |  |  |  |  |  | |  |
| b. working with percentages? | |  |  |  |  |  | |  |
|  | |  |  |  |  |  | |  |
| c. calculating a 15% tip? | |  |  |  |  |  | |  |
|  | |  |  |  |  |  | |  |
| d. figuring out how much a  shirt will cost if it is 25% off? | |  |  |  |  |  | |  |

1. **For each of the following questions, please check the box that best describes your answer.**
   1. When reading the newspaper, how **helpful** do you find tables and graphs that are parts of a story?

| Not at all helpful |  |  |  |  | Extremely helpful |
| --- | --- | --- | --- | --- | --- |
|  |  |  |  |  |  |

- 1. When people tell you the chance of something happening, do you prefer that they use **words** ("it rarely happens”) or **numbers** ("there's a 1% chance")?

| Always prefer  words |  |  |  |  | Always prefer numbers |
| --- | --- | --- | --- | --- | --- |
|  |  |  |  |  |  |

- 1. When you hear a weather forecast, do you prefer predictions using **percentages** (e.g., "there will be a 20% chance of rain today") or predictions using only **words** (e.g., "there is a small chance of rain today")?

| Always prefer percentages |  |  |  |  | Always prefer  words |
| --- | --- | --- | --- | --- | --- |
|  |  |  |  |  |  |

- 1. How **often** do you find numerical information to be useful?

| Never |  |  |  |  | Very often |
| --- | --- | --- | --- | --- | --- |
|  |  |  |  |  |  |

1. How **often** do you have someone help you read instructions, pamphlets, or other written material from your doctor of pharmacy?

| Never | Occasionally | Sometimes | Often | Always |
| --- | --- | --- | --- | --- |
|  |  |  |  |  |

1. How confident are you at filling out medical forms by yourself?

| Never | Occasionally | Sometimes | Often | Always |
| --- | --- | --- | --- | --- |
|  |  |  |  |  |

1. How **often** do you have problems learning about your medical condition because of difficulty understanding written information?

| Never | Occasionally | Sometimes | Often | Always |
| --- | --- | --- | --- | --- |
|  |  |  |  |  |

1. During visits where a decision is made with a clinician in the emergency department, I am most comfortable when. . .

I make decisions about my health care.


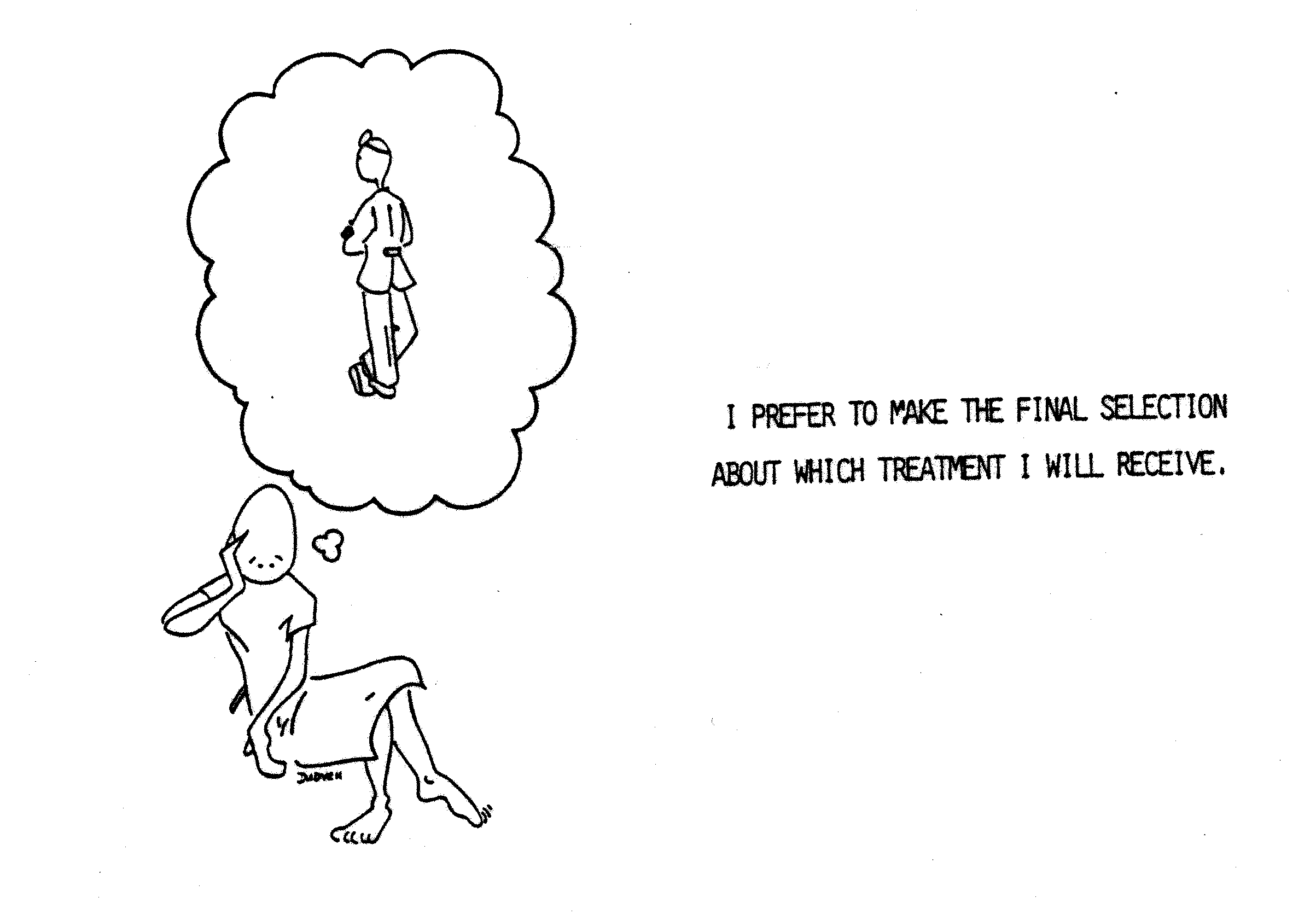


I make decisions about my health care after seriously considering the clinician's opinion.

The clinician makes decisions about my health care, after seriously considering my opinion.

The clinician and I share responsibility for making decisions about my health care.

The clinician makes decisions about my health care.


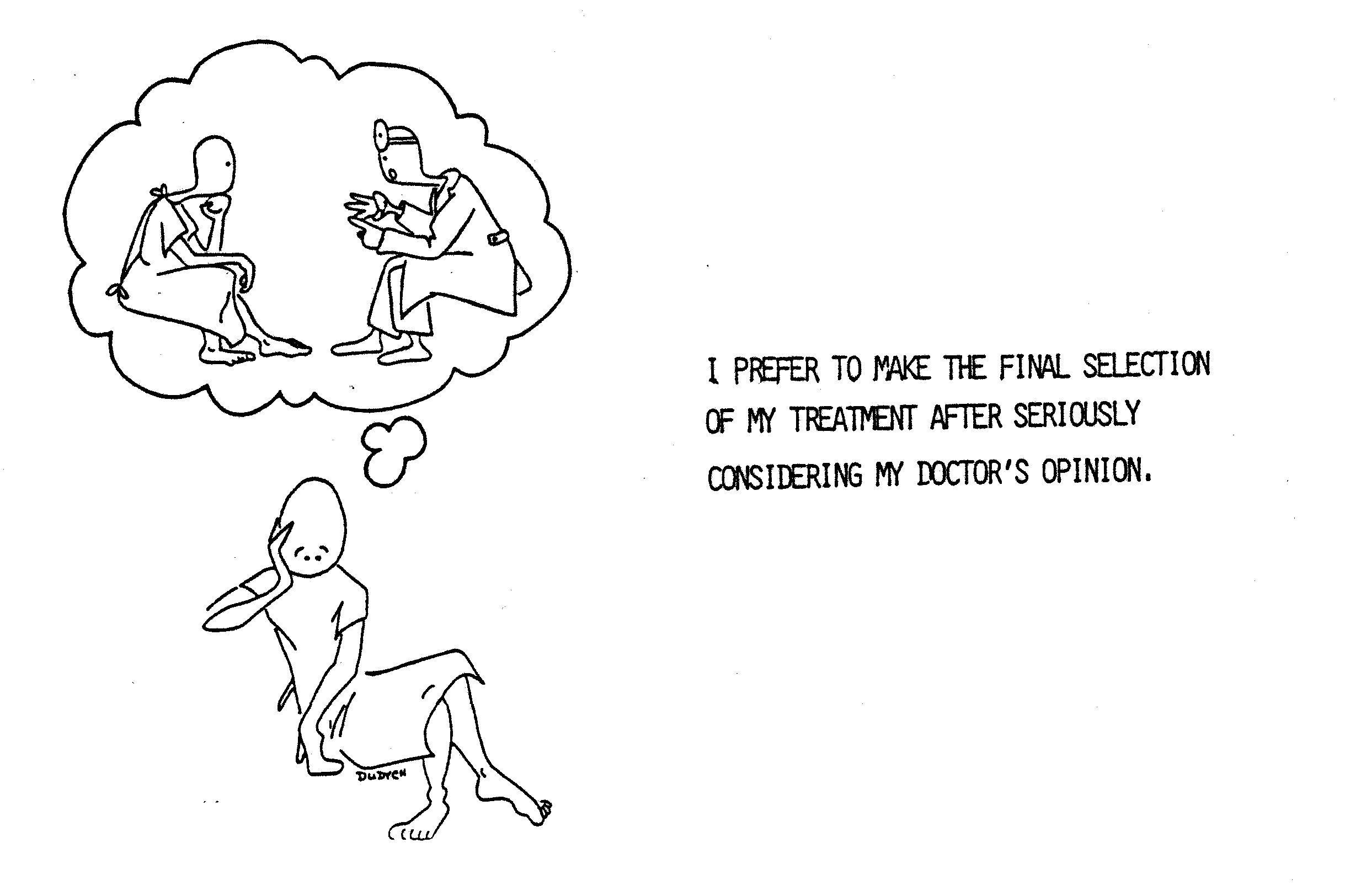

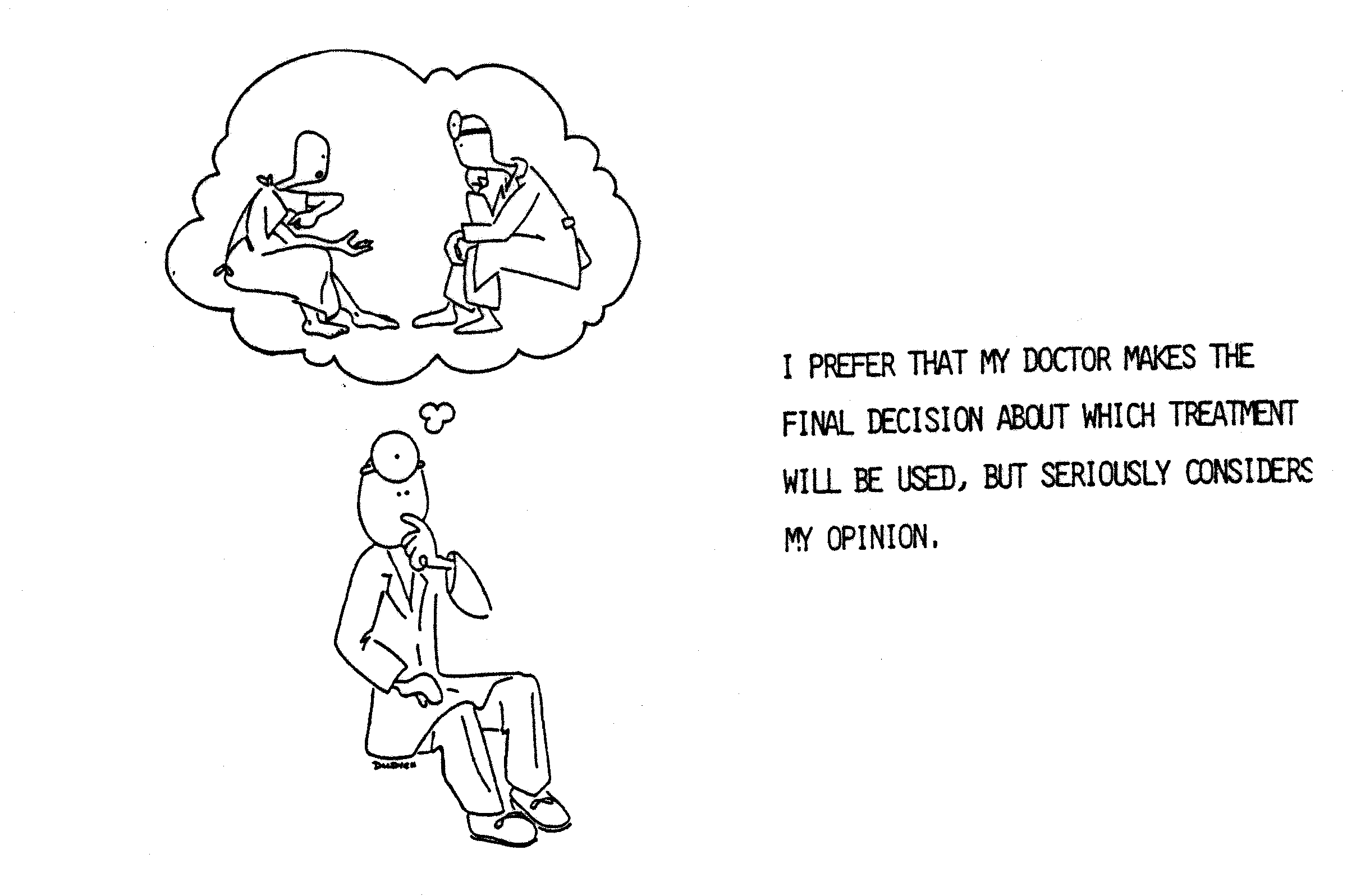

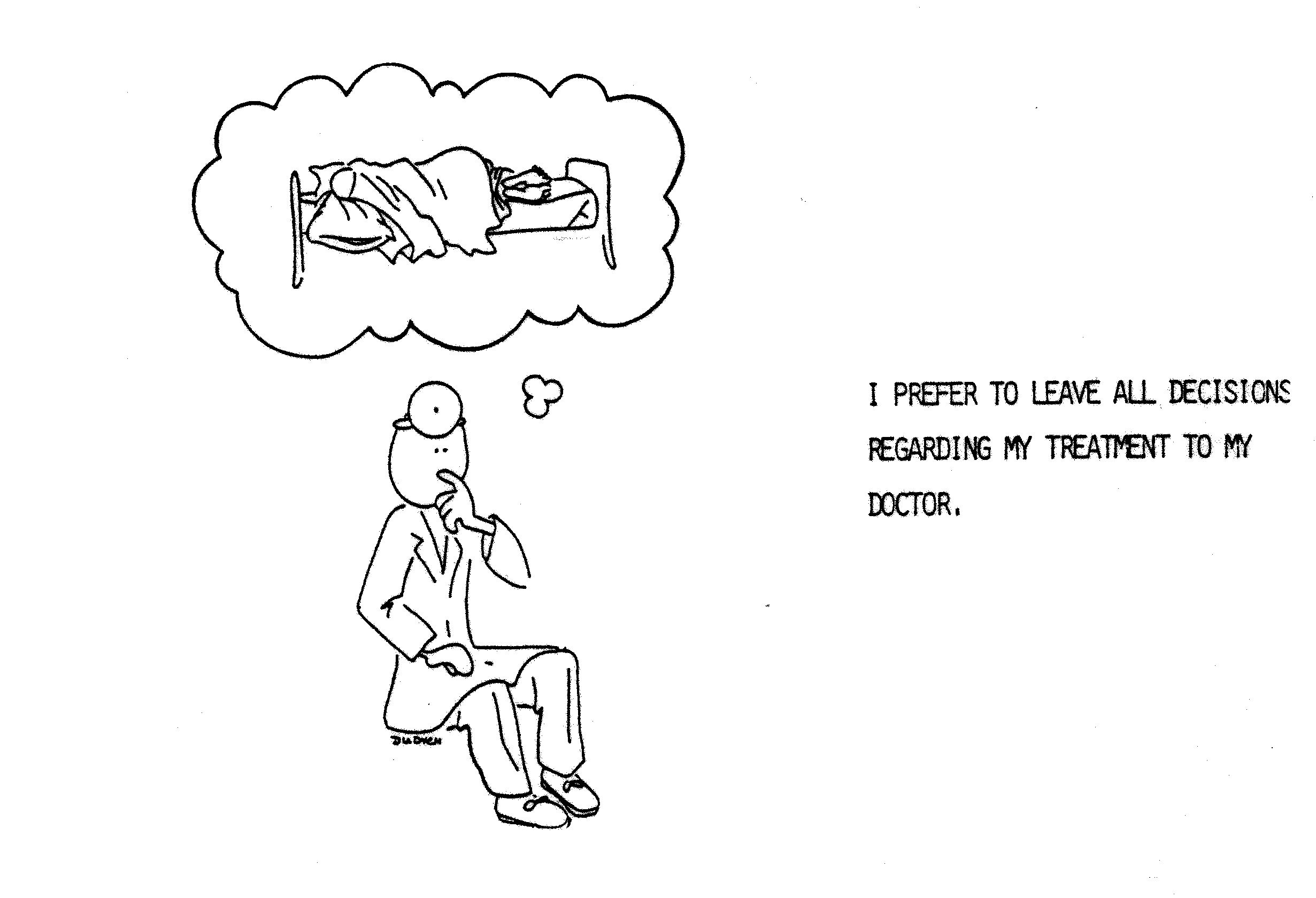

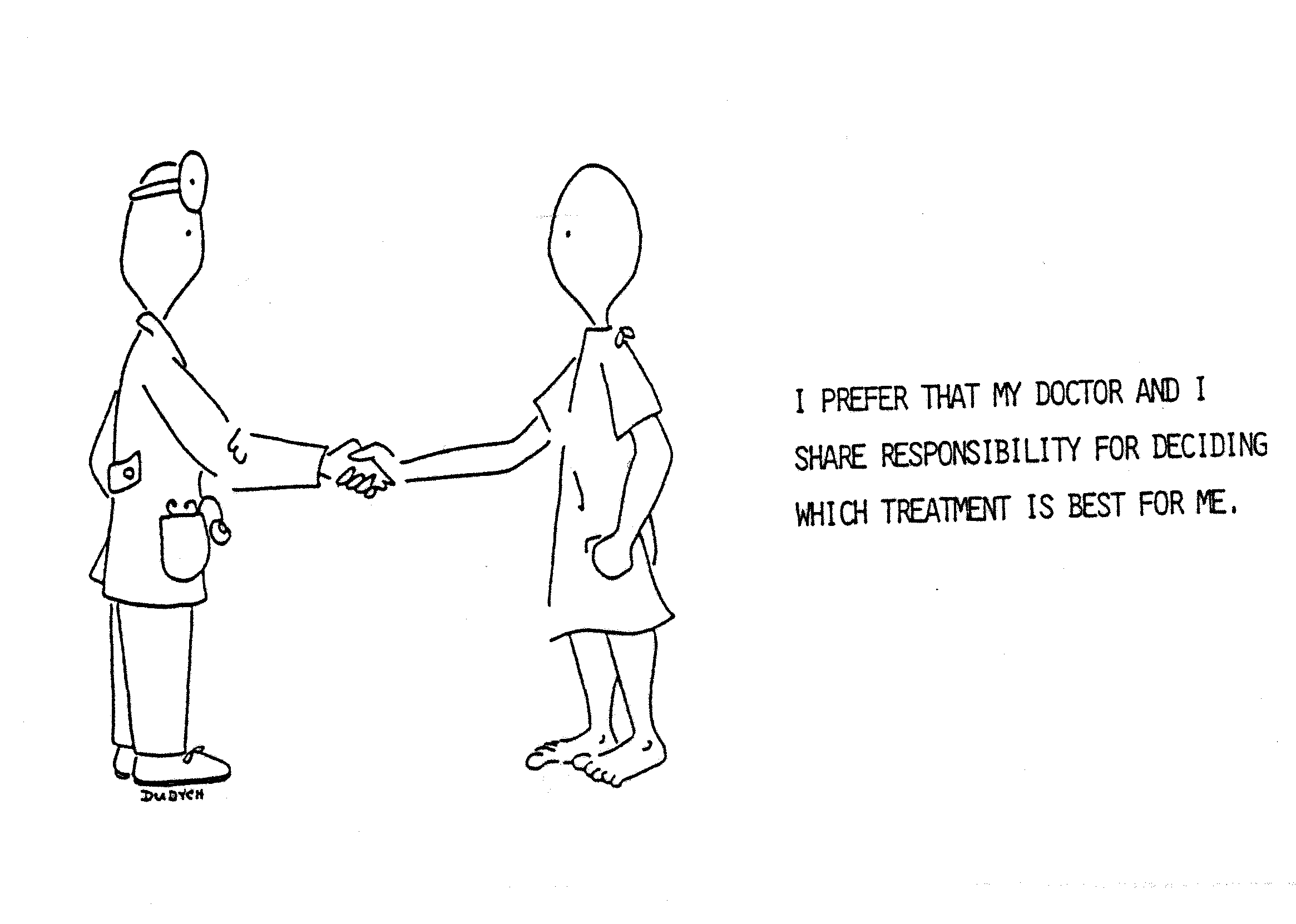


| a. | b. | c.cc. | d. | e. |
| --- | --- | --- | --- | --- |

1. What is the highest level of schooling you have completed?

|  | Some high school or less |
| --- | --- |
|  | High school graduate or GED |
|  | Some college or associate's degree (including community college) or vocational technical, or business school degree |
|  | Four-year college graduate (bachelor's degree) |
|  | Graduate or professional school degree |
|  | Other, please specify:_______________________________________ |

1. Which of the following categories best describes your household income last year?

|  | Less than $20,000 |
| --- | --- |
|  | $20,000 to $29,999 |
|  | $30,000 to $39,999 |
|  | $40,000 to 59,999 |
|  | $60,000 to 79,999 |
|  | $80,000 to 99,999 |
|  | $100,000 or more |

**Thank you for completing this survey!**

**Please return it to the study coordinator.**
