# Supplementary material for: Effectiveness of the Chest Pain Choice decision aid in emergency department patients with low-risk chest pain: study protocol for a multicenter randomized trial
Source: Trials. 2014 May 10;15:166. doi: 10.1186/1745-6215-15-166 (PMC4031497; doi:10.1186/1745-6215-15-166)
Supplement: Additional file 4 — Making Wiser Choices about Chest Pain: post encounter survey. [file 1745-6215-15-166-S4.docx]

Patient ID:

Making Wiser Choices about Chest Pain

Post Encounter Survey


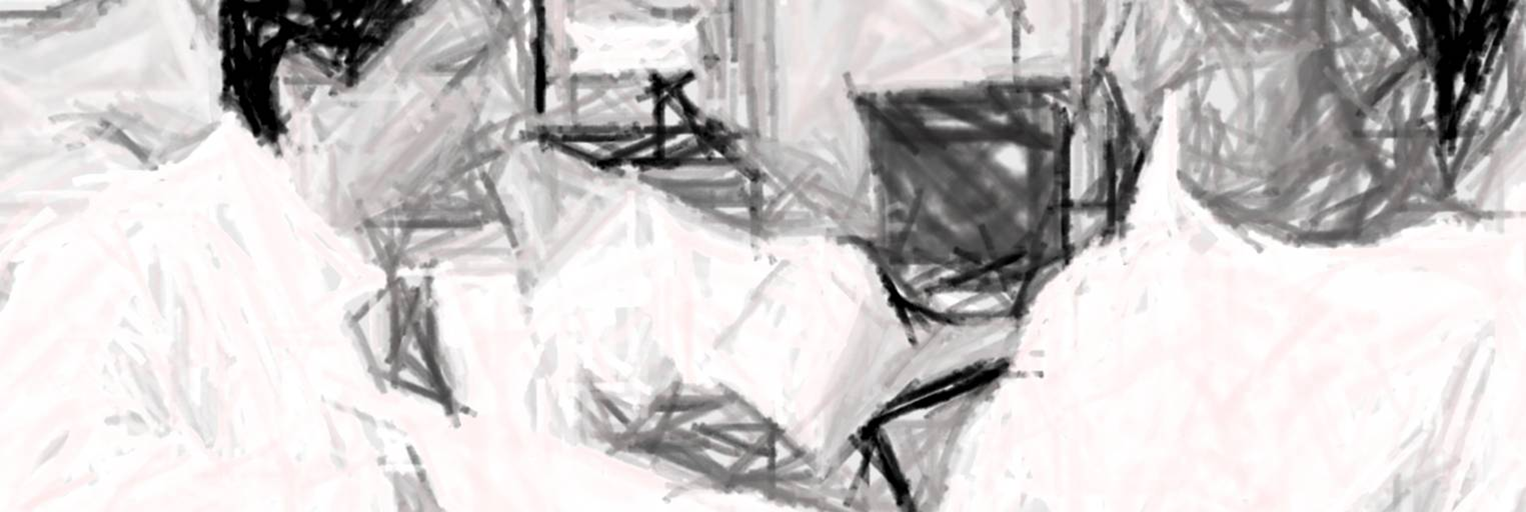


Patient Survey

Today’s Date: _ _/ _ _ / _ _ _ _

Month Day Year

Thank you for helping with this study. Your answers are very important to us. Please take the time to read and answer each question. Your responses are confidential and your clinician will not see your answers.

1. **Which of the following options of care best describes the decision you made today regarding your chest pain symptoms and your options for care?**

|  | To be admitted to the observation unit and have an urgent cardiac stress test. |
| --- | --- |
|  | To follow-up with a heart doctor within 24-72 hours. |
|  | To follow-up with my own primary care physician at the next available appointment. |
|  | To receive a coronary CTA in the emergency department today. |
|  | To have the emergency doctor make the decision for me. |

1. **The following questions are about the visit you had with your clinician today and the discussion you had about your chest pain symptoms and your options for care. Please mark the best answer to each of these questions by marking an X in the box you select.**
   1. How would you describe the **amount of information** about your chest pain symptoms and options for care during this visit?

| Too little  information | | Just the right  amount of  information | | |  | Too much information |
| --- | --- | --- | --- | --- | --- | --- |
|  |  |  |  |  |  |  |

- 1. How would you describe the **clarity of information** about your chest pain symptoms and options for care during the visit?

| Not clear  at all | | Somewhat  clear | | |  | Extremely  clear |
| --- | --- | --- | --- | --- | --- | --- |
|  |  |  |  |  |  |  |

- 1. How **helpful** was the information about your chest pain symptoms and options for care during the visit?

| Not helpful  at all | | Somewhat  helpful | | |  | Extremely  helpful |
| --- | --- | --- | --- | --- | --- | --- |
|  |  |  |  |  |  |  |

- 1. Would you want to get information about other options for care **in the same way** that you got information about your chest pain symptoms and options for care during the visit?

| Yes, for sure | | Not sure | | |  | No, not at all |
| --- | --- | --- | --- | --- | --- | --- |
|  |  |  |  |  |  |  |

- 1. Would you **recommend the way** that you and your provider shared information about your chest pain symptoms and options for care to other patients?

| Yes, I  strongly  recommend  it | | | Not sure  whether to  recommend  it or not | | | |  | | No, I  strongly recommend  against it | |
| --- | --- | --- | --- | --- | --- | --- | --- | --- | --- | --- |
|  | |  |  |  |  | |  | |  | |
|  | |  |  |  |  | |  | |  | |
| 1. **Below are listed some statements about chest pain and your options of care. Please show whether you think they are true, false, or you are unsure.** | | | | | True | | False | | Unsure |  |
| 1. My current chest pain may indicate possible warning signs of a **FUTURE** heart attack. | | | | |  | |  | |  |  |
| 1. I can follow-up with my own doctor at the next available visit and decide with him or her whether I need additional tests. | | | | |  | |  | |  |  |
| 1. I can go home and have a follow-up visit with a heart specialist (cardiologist) within 24-72 hours for additional tests. | | | | |  | |  | |  |  |
| 1. A stress test evaluation will tell me my risk of having a heart attack now. | | | | |  | |  | |  |  |
| 1. The result of a stress test evaluation can be a false positive and lead to additional testing that is unnecessary. | | | | |  | |  | |  |  |
| 1. Having a stress test now will not lengthen my emergency stay. | | | | |  | |  | |  |  |
| 1. None of the stress tests involve exposure to radiation. | | | | |  | |  | |  |  |
| 1. Radiation may increase my future risk of cancer. | | | | |  | |  | |  |  |

1. **If you take 100 people just like you, how many do you think will develop a heart attack or pre-heart attack within the next 45 days?**

Provide a value of 0-100 or respond ‘I do not know’:

_________________ I do not know.

1. **Thinking about the conversation that you had with your clinician today about your chest pain symptoms and options of care, please mark an x inside the box that best describes your agreement with the following statements.**

|  | Strongly  agree | Agree | Neither  agree nor  disagree | Disagree | Strongly  disagree |
| --- | --- | --- | --- | --- | --- |
| a. I know which options are available to me. . . |  |  |  |  |  |
| b. I know the benefits of each option. . . . . . . . . |  |  |  |  |  |
| c. I know the risks and side effects of each  option. . . . . . . . . . . . . . . . . . . . . . . . . . . . . . . |  |  |  |  |  |
| d. I am clear about which benefits matter most  to me. . . . . . . . . . . . . . . . . . . . . . . . . . . . . . . . |  |  |  |  |  |
| e. I am clear about which risks and side effects  matter most to me. . . . . . . . . . . . . . . . . . . . . . |  |  |  |  |  |
| f. I am clear about which is more important to  me (the benefits or the risks and side effects) |  |  |  |  |  |
| g. I have enough support from others to make a  choice . . . . . . . . . . . . . . . . . . . . . . . . . . . . . . |  |  |  |  |  |
| h. I am choosing without pressure from others. |  |  |  |  |  |
| i. I have enough advice to make a choice. . . . . . |  |  |  |  |  |
| j. I am clear about the best choice for me. . . . . . |  |  |  |  |  |
| k. I feel sure about what to choose. . . . . . . . . . . |  |  |  |  |  |
| l. This choice is easy for me to make. . . . . . . . . |  |  |  |  |  |
| m. I feel I have made an informed choice. . . . . |  |  |  |  |  |
| n. My choice shows what is important to me. . . |  |  |  |  |  |
| o. I expect to stick with my choice. . . . . . . . . . . |  |  |  |  |  |
| p. I am satisfied with my choice . . . . . . . . . . . . |  |  |  |  |  |

1. **How much do you trust the provider who discussed your chest pain symptoms and your options for care during your visit today to:**

|  | Trust completely | Trust mostly | Trust somewhat | Trust a little | Trust not at all |
| --- | --- | --- | --- | --- | --- |
| a. Always tell you the truth. . . . . . . . . . . . . . . |  |  |  |  |  |
| b. Provide you with accurate, up-to-date,  medical information. . . . . . . . . . . . . . . . . . . |  |  |  |  |  |
| c. Make it easy for you to bring up a prior  discussion about your condition and  discuss it again. . . . . . . . . . . . . . . . . . . . . . . |  |  |  |  |  |
| d. Make excellent medical judgments on your  behalf. . . . . . . . . . . . . . . . . . . . . . . . . . . . . . |  |  |  |  |  |
| e. Do everything medically that should be  done in order to ensure the best possible  result. . . . . . . . . . . . . . . . . . . . . . . . . . . . . . . |  |  |  |  |  |
| f. Tell you when you could benefit from  seeing a specialist. . . . . . . . . . . . . . . . . . . . . |  |  |  |  |  |
| g. Tell you if a mistake was made about your  treatment. . . . . . . . . . . . . . . . . . . . . . . . . . . |  |  |  |  |  |
| h. Put your medical needs above all other  considerations, including cost. . . . . . . . . . . |  |  |  |  |  |
| i. Listen well so he/she understands your  needs and concerns. . . . . . . . . . . . . . . . . . . . |  |  |  |  |  |
| j. Never pretend to know things when he/she  is not sure. . . . . . . . . . . . . . . . . . . . . . . . . . . |  |  |  |  |  |

1. **Thinking about the decision you made today about your chest pain symptoms and your options for care, would your decision be different if your care was free (no cost to you)?**

|  | Yes |
| --- | --- |
|  | No |

1. **Sometimes people need help completing surveys. Please indicate who answered the majority of the questions in this booklet. (Mark one.)**

|  | Myself |
| --- | --- |
|  | My spouse |
|  | Another family or household member |
|  | Friend |
|  | Emergency room staff |
|  | Research nurse |
|  | Other, please specify: _____________________________________ |

1. **What were the factors that influenced your decision to obtain or not to obtain further cardiac testing?**

___________________________________________________________________

**10. What was the main factor that influenced your decision today?**

___________________________________________________________________

**Thank you for completing the survey!**

**Please return it to the study coordinator.**
