# Supplementary material for: Effectiveness of the Chest Pain Choice decision aid in emergency department patients with low-risk chest pain: study protocol for a multicenter randomized trial
Source: Trials. 2014 May 10;15:166. doi: 10.1186/1745-6215-15-166 (PMC4031497; doi:10.1186/1745-6215-15-166)
Supplement: Additional file 5 — Chest Pain Choice Trial: 45-day follow-up. [file 1745-6215-15-166-S5.docx]

**Chest Pain Choice Trial: 45-day follow-up**

**
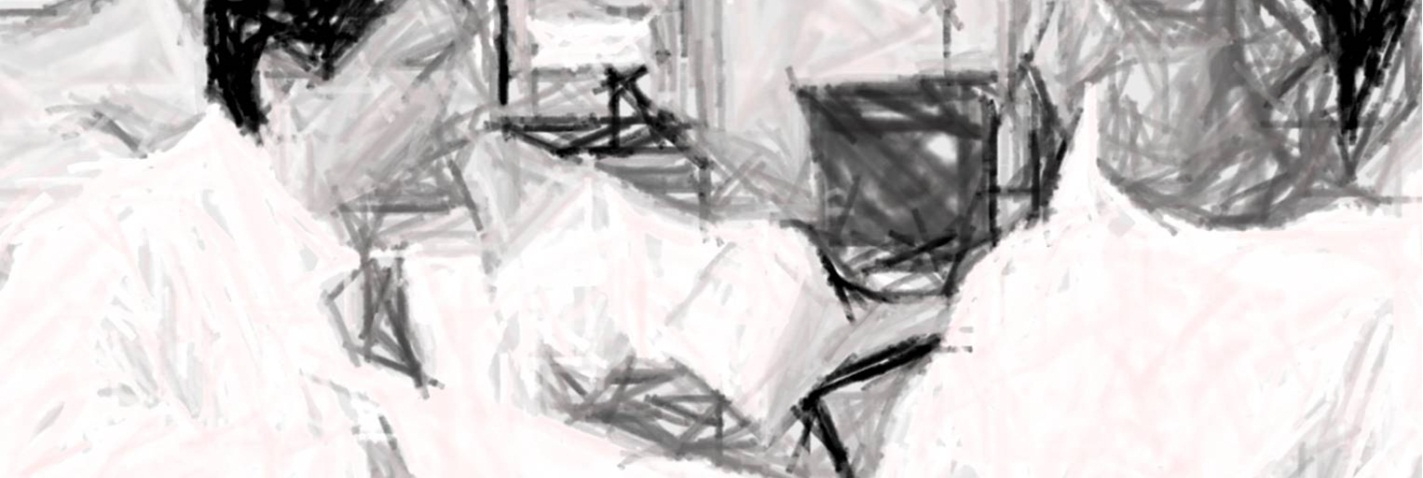
**

Hello, my name is ______________ and I am calling from the Emergency Department at (hospital). May I please speak to (patient name) ____________________?

On (date of ED visit) _______________when you came to the Emergency Department with chest pain, you enrolled in the Chest Pain Choice Study. At that time, we explained we would be calling you in about 45 days, if you remember, we also gave you a health care use diary to record any health care services used after emergency department discharge. So today I'm calling to ask you a few questions regarding what has happened in the 45 days since your visit. Understand that should you choose not to answer these questions that your current and future care at (hospital) will not be affected by whether or not you participate. Specifically, your care will not be jeopardized if you choose not to answer the questions.

Is this alright with you? O Yes O No

Please see the 45 day patient follow-up flow to record contacts/attempted contacts made.

Dead? O Yes O No

Lost to follow-up? O Yes O No

Refused? O Yes O No

1. Since your initial Emergency Department visit, have you been re-admitted to the hospital?

O Yes O No

1. If yes, what was the name of the hospital, the reason for admission, the date of admission, and the date of discharge?

| **Name of Hospital** | **Reason for Admission** | **Date of Admission** | **Date of Discharge** |
| --- | --- | --- | --- |
|  |  |  |  |
|  |  |  |  |
|  |  |  |  |

1. Since your initial Emergency Department visit, have you returned to the Emergency Department?

O Yes O No

1. If yes, what was the name of the hospital, the reason for the visit, and the date of each visit?

| **Name of Hospital** | **Reason for visit** | **Date of Visit** |
| --- | --- | --- |
|  |  |  |
|  |  |  |
|  |  |  |

1. Since your initial Emergency Department visit, have you had an office visit with a physician?

O Yes O No

1. If yes, what was the name of the clinic or physician office, type of physician, and date of each visit?

| **Name of Clinic or Physician Office** | **Reason for Visit** | **Type of Physician (Please Check)** | **Date of Visit** |
| --- | --- | --- | --- |
|  |  | ☐ Primary Care Physician  ☐ Cardiologist  ☐ Other |  |
|  |  | ☐ Primary Care Physician  ☐ Cardiologist  ☐ Other |  |
|  |  | ☐ Primary Care Physician  ☐ Cardiologist  ☐ Other |  |

1. Since your initial Emergency Department visit, have you had any blood testing, x-rays, CT’s, or cardiac (heart) stress testing?

O Yes O No

1. If yes, what was the type of test, name of the hospital or clinic, the date of the test, and the test result?

| **Type of Test** | **Name of Hospital or Clinic** | **Date of Test** |
| --- | --- | --- |
| ☐ Blood Test  ☐ CT (Computed Tomography)  ☐ Cardiac (Heart) Stress Test  ☐ Other (please describe):__________________________ |  |  |
| ☐ Blood Test  ☐ CT (Computed Tomography)  ☐ Cardiac (Heart) Stress Test  ☐ Other (please describe):________________________ |  |  |
| ☐ Blood Test  ☐ CT (Computed Tomography)  ☐ Cardiac (Heart) Stress Test  ☐ Other (please describe):__________________________ |  |  |

1. Since your initial Emergency Department visit, have you undergone angioplasty or stent placement in your coronary arteries or coronary artery bypass graft surgery?

O Yes O No

1. If yes, what procedure, and what was the date of that procedure?

O Angioplasty/stent placement (d/m/y)___/___/___

O CABG (d/m/y)___/___/___

1. Since your Emergency Department visit, did a clinician tell you that you had a myocardial infarction or heart attack?

O Yes O No
