# Supplementary material for: Effectiveness of the Chest Pain Choice decision aid in emergency department patients with low-risk chest pain: study protocol for a multicenter randomized trial
Source: Trials. 2014 May 10;15:166. doi: 10.1186/1745-6215-15-166 (PMC4031497; doi:10.1186/1745-6215-15-166)
Supplement: Additional file 6 — Chest Pain Study: use of healthcare services diary. [file 1745-6215-15-166-S6.docx]

Chest Pain Study

Use of Healthcare Services

Diary


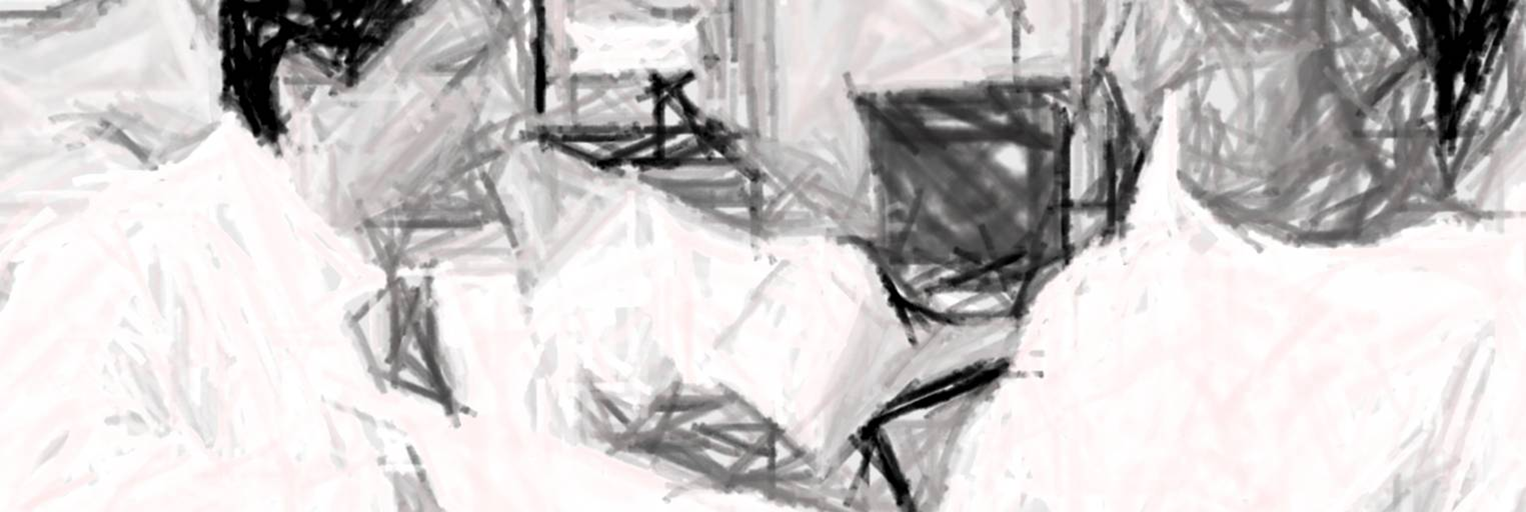


You were recently enrolled in the **Chest Pain Study.** As part of this study, we would like to understand your use of healthcare services in the **45-days AFTER discharge** from the emergency department.

This *Diary* is for you to keep, in order that you may have a record of events related to your health from the time of your discharge from the emergency department.

In addition, as you may be aware, a study researcher will contact you in about 45-days after you joined the study to ask you about events related to your health.

To keep track of these events you may find this *Diary* will help you answer the questions. If you require additional space to record details of health service use, please use the sheet entitled *Events Diary -additional information* that is included.

**Hospital Admissions**

If you are admitted to a hospital, please write the **name** of the hospital, **reason** for being admitted and **dates** of each admission and discharge. Use one line for each hospital admission.

| **Name of Hospital** | **Reason for Admission** | **Date of Admission** | **Date of Discharge** |
| --- | --- | --- | --- |
|  |  |  |  |
|  |  |  |  |
|  |  |  |  |
|  |  |  |  |
|  |  |  |  |

**Emergency Department Visits**

If you visit an emergency department, please write the **name** of the hospital, **reason** for the visit and **date** of each visit. Use one line for each Emergency Department visit.

| **Name of Hospital** | **Reason for visit** | **Date of Visit** |
| --- | --- | --- |
|  |  |  |
|  |  |  |
|  |  |  |
|  |  |  |
|  |  |  |

**Physician Office Visit**

If you visit a physician office, please write the **name** of the health care facility, **reason** for the visit, **type** of physician and **date** of each visit. Use one line for each office visit.

| **Name of Clinic or Physician Office** | **Reason for Visit** | **Type of Physician (Please Check)** | **Date of Visit** |
| --- | --- | --- | --- |
|  |  | ☐ Primary Care Physician  ☐ Cardiologist  ☐ Other |  |
|  |  | ☐ Primary Care Physician  ☐ Cardiologist  ☐ Other |  |
|  |  | ☐ Primary Care Physician  ☐ Cardiologist  ☐ Other |  |
|  |  | ☐ Primary Care Physician  ☐ Cardiologist  ☐ Other |  |
|  |  | ☐ Primary Care Physician  ☐ Cardiologist  ☐ Other |  |

**Testing**

In the next section, we would like you to document any testing that you receive in the 45-days after leaving the emergency department. This testing includes **blood tests, x-rays, CTs, or cardiac (heart) stress testing**. Please indicate the type of test, hospital or clinic where the test was conducted, and date of test

| **Type of Test** | **Name of Hospital or Clinic** | **Date of Test** |
| --- | --- | --- |
| ☐ Blood Test  ☐ CT (Computed Tomography)  ☐ Cardiac (Heart) Stress Test  ☐ Other (please describe):__________________________ |  |  |
| ☐ Blood Test  ☐ CT (Computed Tomography)  ☐ Cardiac (Heart) Stress Test  ☐ Other (please describe):__________________________ |  |  |
| ☐ Blood Test  ☐ CT (Computed Tomography)  ☐ Cardiac (Heart) Stress Test  ☐ Other (please describe):__________________________ |  |  |
| ☐ Blood Test  ☐ CT (Computed Tomography)  ☐ Cardiac (Heart) Stress Test  ☐ Other (please describe):__________________________ |  |  |
| ☐ Blood Test  ☐ CT (Computed Tomography)  ☐ Cardiac (Heart) Stress Test  ☐ Other (please describe):__________________________ |  |  |

***Events Diary - Additional Information***
