# Supplementary material for: Effectiveness of the Chest Pain Choice decision aid in emergency department patients with low-risk chest pain: study protocol for a multicenter randomized trial
Source: Trials. 2014 May 10;15:166. doi: 10.1186/1745-6215-15-166 (PMC4031497; doi:10.1186/1745-6215-15-166)
Supplement: Additional file 7 — Making wiser choices about Chest Pain: clinician post encounter survey. [file 1745-6215-15-166-S7.doc]

Patient Study ID:

_ _ _ _ _ _ _ _ _

Clinician’s Initials:

_______________

Today’s Date:

_ _ / _ _ / _ _ _ _

Month Day Year

Making wiser choices about Chest Pain:

Clinician Post Encounter Survey


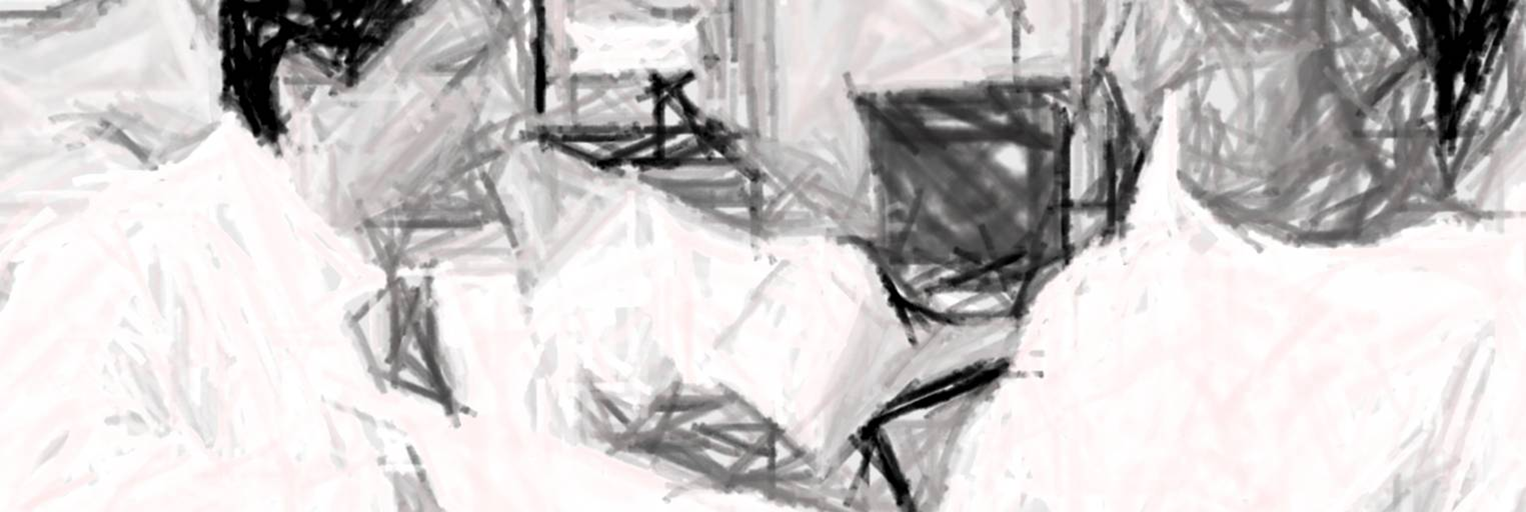


1. **In the clinical encounter where the decision was made with this patient, . . .**

| ❑ | I made the decision on my own. |
| --- | --- |
| ❑ | I made the decision after seriously considering the patient’s opinion. |
| ❑ | The patient and I shared the responsibility for making the decision after considering both of our opinions. |
| ❑ | The patient made the decision after seriously considering my opinion. |
| ❑ | The patient made the decision on his/her own. |

1. **You gave information about acute coronary syndrome, the patient’s risk for acute coronary syndrome (ACS), and their diagnostic options during this visit. How *helpful* do you think this information was to the patient?**

| ❑ | ❑ | ❑ | ❑ | ❑ | ❑ | ❑ |
| --- | --- | --- | --- | --- | --- | --- |
| Not helpful at all |  |  | Somewhat helpful |  |  | Extremely helpful |

1. **Would you want to present information about other diagnostic choices in the same way that you presented information about ACS during this visit?**

| ❑ | ❑ | ❑ | ❑ | ❑ | ❑ | ❑ |
| --- | --- | --- | --- | --- | --- | --- |
| Yes, for sure |  |  | Not sure |  |  | No, not at all |

1. **Would you *recommend* to other providers the way that you presented information about ACS, the patient’s risk for ACS, and their diagnostic options during this visit?**

| ❑ | ❑ | ❑ | ❑ | ❑ | ❑ | ❑ |
| --- | --- | --- | --- | --- | --- | --- |
| Yes, I would strongly recommend it |  |  | Not sure whether to recommend it or not |  |  | No, I would strongly recommend against it |

1. **Thinking about the conversation you had with your patient today about ACS, the patient’s risk for ACS, and their diagnostic options, please place an “X” inside the box that best describes your agreement with the following statements.**

|  | Strongly agree | Agree | Neither agree nor disagree | Disagree | Strongly disagree |
| --- | --- | --- | --- | --- | --- |
|  |  |  |  |  |  |
| 1. I feel the patient has made an informed choice. . . . . . . . . . . . . . | ❑ | ❑ | ❑ | ❑ | ❑ |
| 1. The patient’s decision shows what is important to him/her. . . . . . . . . | ❑ | ❑ | ❑ | ❑ | ❑ |
| 1. I expect the patient to stick with his/her decision. . . . . . . . . . . . . . . | ❑ | ❑ | ❑ | ❑ | ❑ |
| 1. I think the patient is satisfied with his/her decision. . . . . . . . . . . . . . . | ❑ | ❑ | ❑ | ❑ | ❑ |

**Thank you for completing the survey and participating in the trial. Please return the survey to the study coordinator.**
